# Experiences of General Practitioners and Practice Assistants during the Influenza A(H1N1) Pandemic in the Netherlands: A Cross-Sectional Survey

**DOI:** 10.1371/journal.pone.0135666

**Published:** 2015-08-27

**Authors:** Christel E. van Dijk, Mariette Hooiveld, Anne Jentink, Leslie D. Isken, Aura Timen, C. Joris Yzermans

**Affiliations:** 1 NIVEL, Netherlands Institute for Health Services Research, Utrecht, The Netherlands; 2 Preparedness and Response Unit, Centre for Infectious Disease Control, National Institute for Public Health and the Environment (RIVM), Bilthoven, The Netherlands; Health Protection Agency, UNITED KINGDOM

## Abstract

**Objectives:**

Since few pandemics have occurred since the Spanish influenza pandemic, we should learn from every (mild) pandemic that occurs. The objective of this study was to report on general practitioners’ and practice assistants’ acceptance of the chosen national policy, and experiences in the Netherlands during the influenza A(H1N1)pdm09 pandemic.

**Methods:**

Data on experience and acceptance of the chosen national policy were obtained by structured questionnaires for general practitioners (n = 372) and practice assistants (n = 503) in April 2010.

**Results:**

The primary policy chosen for general practice was not always accepted and complied with by general practitioners, although the communication (of changes) and collaboration with involved organisations were rated as positive. In particular, the advised personal protective measures were difficult to implement in daily work and thus not executed by 44% of general practitioners. Half of the general practitioners were not satisfied with the patient information provided by the government. The influenza A(H1N1) pandemic highly impacted on general practitioners’ and practice assistants’ workloads, which was not always deemed to be adequately compensated.

**Discussion:**

Involvement of general practitioners in future infectious disease outbreaks is essential. This study addresses issues in the pandemic policy which might be critical in a more severe pandemic.

## Introduction

New human-transmissible viruses may present in the near future, which could result in a pandemic with catastrophic consequences. The current outbreak of Ebola in West Africa underlines this risk [1]. Since few pandemics have occurred since the Spanish influenza pandemic, we should learn from every pandemic that occurs. The 2009 influenza A(H1N1)pdm09 pandemic, although mild, provides the opportunity to test the chosen pandemic policy [2]. The aim of this study was to report on the acceptance of general practitioners (GPs) and practice assistants of the chosen national policy and their experiences in the Netherlands during the influenza A(H1N1) pandemic.

Previous research has shown that the influenza A(H1N1) pandemic had an impact on the workload of health care workers. One study addressed the impact of the influenza A(H1N1) pandemic on frontline public health workers employed by the communicable disease departments of public health services in the Netherlands, and showed that overall, most public health workers complied with control measures and their workload was considered high during the first months of the pandemic [3]. Several international studies have evaluated the impact of the influenza A(H1N1) pandemic on health care workers and their acceptance of the chosen policy. In general, the workload of health care workers increased during the pandemic, which resulted in personal stress and fatigue as well as less time for non-influenza patients [4–7]. Personal protective equipment was generally tolerated, but deemed uncomfortable to wear over prolonged periods [4–5]. In some cases, isolation of suspected or infected patients was considered difficult in the current practice circumstances [4]. Changes in the management of suspected and infected patients resulted in confusion and were not always communicated well [6,8]. Media coverage was considered sensational and resulted in unnecessary health care demands [4].Previous research has predominantly focused on frontline public health workers and health personnel in hospitals. However, GPs and their practice assistants have also played an important role in the influenza A(H1N1) pandemic. In the Netherlands, as in the United Kingdom, GPs act as gatekeepers for specialised, secondary care and form the first point of contact for patients in the health care system [9]. Patients with manifestation of influenza-like illnesses and concerns regarding influenza A(H1N1) most likely present to a GP first. In general practice, practice assistants play an important role in assessing telephone requests and informing patients about vaccination. This evaluation of GPs’ and practice assistants’ acceptance of the chosen policy and experiences during the influenza A(H1N1) pandemic provides the opportunity to examine the impact of the influenza A(H1N1) pandemic and management within general practice, with the aim of addressing issues that might be more critical in a more severe pandemic.

### General Policy during the Pandemic, and the Timeline of Recommendations regarding the Influenza A(H1N1) Pandemic in General Practice in the Netherlands

In the Netherlands, the municipal health services (MHS) are responsible for infectious disease control in their area. Under certain circumstances, the Ministry of Health can decide to take over regional responsibilities by giving a certain disease a specific mandatory status. Infectious diseases with a specific mandatory status are notifiable (by GPs) to the regional public health service in the Netherlands. The extent of the outbreak control measurements are nationally aligned and thereafter implemented and communicated by the MHS. During an outbreak, all involved actors work together in an outbreak management team and in managerial coordination meetings [10].

**Fig 1:**
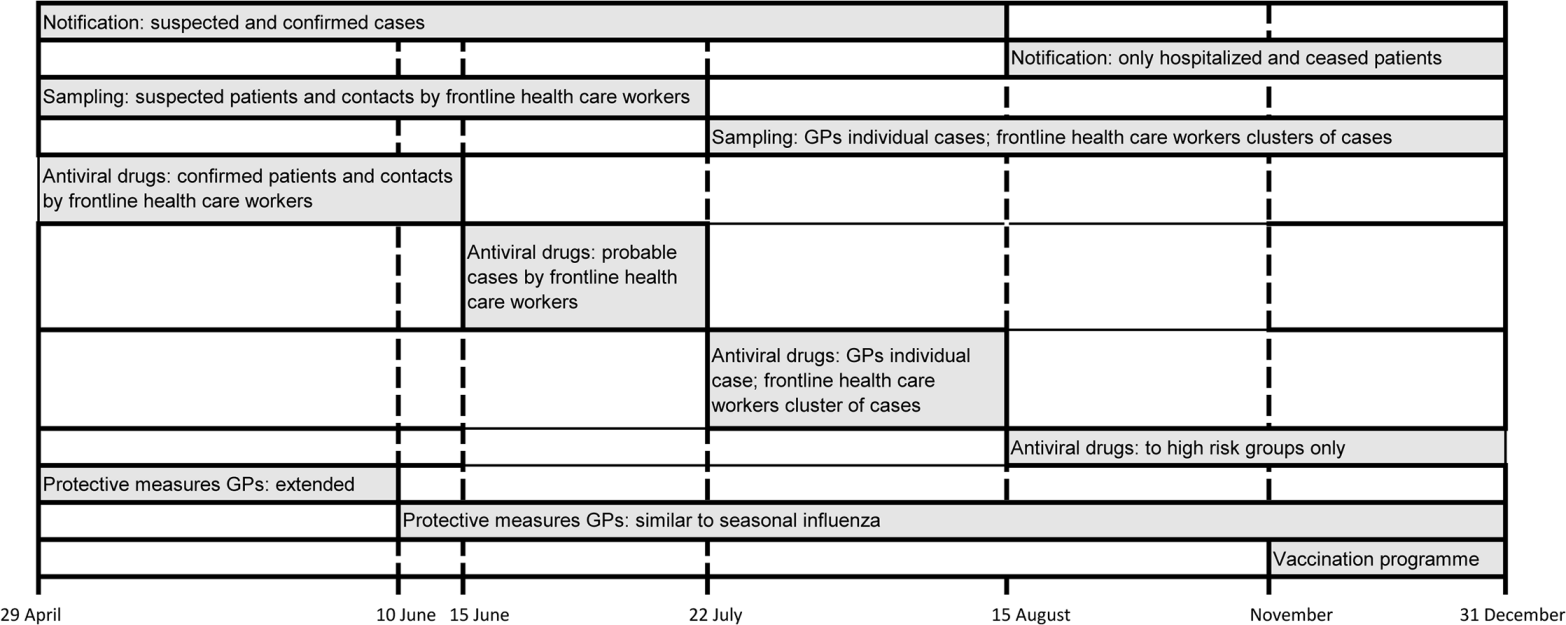
Timeline of recommendations regarding the influenza A(H1N1) pandemic in general practice in the Netherlands, 29 April-31 December 2009.

The timeline of recommendations regarding the influenza A(H1N1) pandemic in general practice in the period 29 April-31 December 2009 is shown in Fig 1. The influenza A(H1N1)pdm09 virus became notifiable on April 29^th^ of 2009. In the influenza A(H1N1) pandemic period, the MHSs informed patients and GPs with the aid of information leaflets provided by the National Institute for Public Health and Environment (RIVM), commissioned by the Ministry of Health. The RIVM also informed the national press, while regional MHSs covered local media. Initially, policy aimed at virus containment. Frontline public health workers of MHSs took samples from all patients with suspected influenza A(H1N1) viral infection, as well as from their contacts. Antiviral drugs were given by frontline public health workers to all confirmed patients and their contacts. From 15 June 2009, antiviral drugs were also administrated to probable cases [3]. GPs were advised to wear gloves, face masks, goggles and disposable aprons upon contact with suspected patients, to disinfect hands after contact with patients and to provide face masks to suspected patients. After contact with suspected patients, GPs had to dispose of gloves, masks and aprons, and clean instruments, materials and the consultation room with 70% alcohol [11].

As the pandemic proved to have a mild progress, on 10 June the infection prevention policy for the influenza A(H1N1) virus was changed to a policy similar to that for a normal seasonal influenza: masks and gloves only. After 22 July 2009, GPs were responsible for assessing and managing individual cases; clusters of cases were still managed by frontline public health workers. From 15 August, the notification procedure of the influenza A(H1N1) virus infected patients was adjusted. Only hospitalised and deceased patients due to the influenza A(H1N1) virus were mandatorily notified to the public health service [12]. By then, antiviral drugs were given to patients belonging to high-risk groups only.

On 23 October 2009, the incidence of patients with influenza-like illnesses was reported above baseline level, which resulted in the status of an ‘epidemic’ of the new influenza A(H1N1) virus. In November 2009, a vaccination programme started for risk groups [13]. GPs had to administrate two vaccine doses for the influenza A(H1N1) virus besides the seasonal influenza vaccination.

## Materials and Methods

### Study population

A sample of 850 GPs (9.5% of the Dutch GP population) and one of their practice assistants were invited to participate in the study. The sample was randomly drawn from the Dutch GP registration and was representative of the Dutch GP population with respect to age, sex, type of practice (single-handed, duo, group or health care centre), and urbanisation level of the practice location [14]. According to the Dutch legislation, retrospective surveys do not require ethical approval. Information was anonymised and de-identified prior to analysis.

### Questionnaire

Structured questionnaires for GPs and practice assistants were developed based on a literature study on experiences of health care workers during the influenza A(H1N1) pandemic, and on the results of four in-depth interviews with GPs who had worked during the influenza pandemic in the Netherlands. The questionnaire was tested in a pilot study to assess its feasibility and completeness involving GPs and researchers. Based on results of the pilot study, final questionnaires were adapted and sent to general practices in April 2010. Participants had the choice to fill in an anonymous written or online questionnaire. The questionnaires addressed various time spans during the influenza A(H1N1) pandemic, which were presented in chronological order. Participants were asked to rate their acceptance using a 4-point (strongly agree, agree, disagree, strongly disagree) or 5-point Likert scale (for collaboration questions only: excellent, good, neutral, poor, very poor).

### General practitioners

The questionnaire addressed the period from April until December 2009. Several domains were covered: information provided by health care authorities (April-December), collaboration with various organisations (April-December), policy on protective measures (April-22 June), mandatory notification (April-15 August), antiviral drugs provision by the regional public health service (April-22 July), changes in policy on infection prevention and patients’ assessment and management (June-July), notification changes (15 August-December), policy on antiviral drugs for high-risk patients only (15 August-December), and vaccination programme (November).

### Practice assistants

The practice assistants’ questionnaire addressed the periods April-August and October-December 2009. Domains covered were information provision and workload.

### Data analysis

Data were analysed using STATA 12.1. Descriptive statistics were generated. The 4-point Likert scale was recoded into (strongly) agree and (strongly) disagree and the 5-point Likert scale was recoded into excellent/good, neutral and (very) poor, due to a low number of cases in the extreme categories. Non-responder analyses were performed for GPs’ gender, age, function, practice type and the degree of urbanisation of the practice location.

## Results

Of the 850 GPs contacted, 372 completed the questionnaire and 17 GPs were non-eligible since they had not worked as GPs in 2009, resulting in a net response rate of 45%. Completed questionnaires were received from 502 practice assistants, and 15 practice assistants had not worked as assistants in 2009, resulting in a net response rate of 60%. Characteristics of participants are listed in Table 1. Non-responder analyses for GPs showed no significant differences in gender, age, practice type or urbanisation level. However, non-responders were more often employed by another GP rather than being a free entrepreneur (p<0.037). The included GPs were representative of the Dutch GP population with respect to age, sex, type of practice and urbanisation level of the practice location, but not with regard to the function.

**Table 1:** Characteristics of general practitioners and practice assistants.

	General practitioners (n = 372)	General practitioners in the Netherlands 2010(n = 8.921)^$^	Practice assistants (n = 502)
*GP characteristics*			
Gender (female)	146 (39.3%)	39.6%	n.a.
Age (years)			
<40	65 (17.5%)	21.1%	n.a.
40–49	91 (24.5%)	30.4%	n.a.
50–59	167 (44.9%)	38.2%	n.a.
60+	49 (13.2%)	10.3%	n.a.
Function			
Free entrepreneur	356 (95.7%)	87.8%	n.a.
Employed	16 (4.3%)	12.2%	n.a.
*Practice characteristic*			
Practice type			
Single-handed	80 (21.5%)	18%	117 (23.3%)
Duo	106 (28.5%)	28%	141 (28.0%)
Group or health care centre	186 (50.0%)	54%	244 (48.6%)
Urbanisation			
Extremely urbanised	77 (20.7%)	20.0%	102 (20.3%)
Strongly urbanised	111 (29.8%)	27.8%	138 (27.4%)
Moderately urbanised	79 (21.2%)	18.9%	101 (20.1%)
Hardly urbanised	69 (18.6%)	21.8%	103 (20.5%)
Not urbanised	36 (9.7%)	11.6%	58 (11.5%)

^$^Only percentages known; n.a.: not available

### General practitioners

#### Information and collaboration (April-December 2009)

Almost all GPs received information on the influenza A(H1N1) virus via the regional public health service (97%), GP organisations (Dutch College of General Practitioners (NHG), National Association of General Practitioners (LHV); 98%), Ministry of Health (98%) and the National Institute for Public Health and Environment (RIVM; 99%).

GPs were more positive about the information provided to themselves than about the information provided by the government to patients (Table 2). Around two thirds of the GPs experienced the provision of information to themselves as well timed, complete and explicitly formulated, whereas half or less than half of the GPs experienced this for the information provided by the government to patients. Almost all GPs experienced an increased workload due to hectic coverage in the media about the influenza A(H1N1) virus. Most GPs rated the collaboration with the regional public health service hospital and laboratories as good to excellent.

**Table 2:** Policy acceptance and experiences of general practitioners during the influenza A(H1N1) pandemic.

Aspects during the influenza A(H1N1) pandemic	N total	N (strongly) agree	% (strongly) agree (95% CI)
*Information provision (April-December 2009)*			
Information provision to GPs was well timed	365	262	71.8% (67.1–76.4)
Information provision to GPs was complete	364	237	65.1% (60.2–70.0)
Information was explicitly formulated	365	216	59.2% (54.1–64.2)
Information provided by the government for patients was explicit	363	160	44.1% (39.0–49.2)
Information provided by the government for patients was well timed	363	195	53.7% (48.6–58.8)
Information provided by the government for patients was complete	358	145	40.5% (35.4–45.6)
Hectic in the media about the influenza A(H1N1) virus led to increased workload	372	360	96.8% (95.0–98.6)
Additional information about the influenza A(H1N1) could be found when needed	370	334	90.3% (87.3–93.3)
*Protective measures (April-10 June 2009)*			
Clear on when to take protection measure	371	254	68.5% (63.7–73.2)
Personal protection measures were feasible	371	130	35.0% (30.2–39.9)
Personal protection measures were executed	364	205	56.3% (51.2–61.4)
Sufficient personal protection materials were in practice	370	272	73.5% (69.0–78.0)
*Mandatory notification of influenza A(H1N1) virus infections (April-15 August 2009)*			
Clear on which patients needed to be reported	368	253	68.8% (64.0–73.5)
Clear on when patients needed to be reported	368	241	65.5% (60.6–70.3)
Clear on how patients needed to be tested	367	255	69.5% (64.8–74.2)
The notification was useful	354	161	45.5% (40.3–50.7)
The notification was feasible	357	204	57.1% (52.0–62.3)
*Provision of antiviral drugs by the regional public health service (April-22 July 2009)*			
Correct choice that the regional public health service prescribed antiviral drugs instead of the general practitioner	326	186	57.1% (51.7–62.4)
Good collaboration with regional public health service regarding treatment of patients	213	162	76.1% (70.3–81.8)
Did you prescribe antiviral drugs in this period?	355	144	40.6% (35.5–45.7)
*Changes in policy infection prevention and assessment and management of patients (June-July 2009)*			
Policy changes were well communicated	369	221	59.9% (54.9–64.9)
Good that general practitioners became responsible for the sampling of patients	368	254	69.0% (64.3–73.7)
Clear on when to perform diagnostic test on patients	368	207	56.3% (51.2–61.3)
Diagnostic test was feasible	361	227	62.9% (57.9–67.9)
*Changes in notification of influenza A(H1N1) virus infections (15 August-December 2009)*			
Changes in notification or reporting were well communicated	367	294	80.1% (76.0–84.2)
There was sufficient knowledge about these changes	366	308	84.2% (80.4–87.9)
Good decision to limit notification to hospitalised and deceased patients	364	344	94.5% (92.2–96.8)
*Policy on antiviral drugs for high-risk patients only (15 August-December 2009)*			
Recommendation regarding provision of antiviral drugs was well communicated	369	231	62.6% (57.7–67.5)
Clear on which patients to prescribe antiviral drugs for	367	257	70.0% (65.3–74.7)
Advice regarding the prescription of antiviral medicines was complete	368	217	59.0% (53.9–64.0)
*Vaccination programme for influenza A(H1N1) in general practice (November 2009)*			
Clear on who belonged to risk groups for vaccination	369	330	89.4% (86.3–92.6)
During the vaccination rounds, not a lot of time for normal daily work	368	261	70.9% (66.3–75.6)
During the epidemic, tasks were different compared with a regular influenza season	370	272	73.5% (69.0–78.0)
Additional tasks due to vaccination were sufficiently compensated	365	183	50.1% (45.0–55.3)

#### Protective measures (April-10 June 2009)

For two thirds of the GPs, it was clear when to take protective measures, and personal protection material was sufficiently available for three quarters of the GPs. Two thirds of the GPs reported that the personal protection measures were difficult to implement in daily work, and almost half of the GPs stated that they did not execute the personal protective measures.

#### Mandatory notification of influenza A(H1N1) virus infections (April-15 August 2009)

For two thirds of the GPs, it was clear which and when patients needed to be reported to the regional public health services and how patients needed to be tested. Less than half of the GPs experienced the mandatory notification criteria as useful, and 57% of GPs experienced the notification as feasible.

#### Provision of antiviral drugs by the regional public health service (April-22 July 2009)

Fifty-seven per cent of the GPs experienced the choice for the regional public health service to prescribe antiviral drugs instead of GPs as correct, and the collaboration was experienced as good by three quarters of the GPs.

#### Changes in policy regarding infection prevention and assessment and management of patients (June-July 2009)

Sixty per cent of the GPs noted that the changes were well communicated, and 69% of GPs experienced the responsibility for sampling as good. To a substantial part of the GPs, it was not clear when to perform a diagnostic test (44%), and the test was experienced as non-feasible (37%).

#### Changes in notification criteria of influenza A(H1N1) virus infections (15 August-December 2009)

Most GPs agreed with the changes in notification criteria for new influenza A(H1N1) virus infections, and 80% of GPs reported the changes to be communicated well.

#### Policy on antiviral drugs for high-risk patients only (15 August-December 2009)

Around forty per cent of the GPs experienced the recommendation regarding the provision of antiviral drugs as not well communicated, and experienced the advice as incomplete.

#### Vaccination programme influenza A(H1N1) in general practice (November 2009)

To most GPs, it was clear who belonged to the risk groups eligible for vaccination. Almost three quarters of the GPs reported less available time for normal daily work during the vaccination round, and different tasks compared with a regular influenza season during the epidemic. Half of the GPs reported the compensation for additional tasks during the vaccination round as insufficient.

#### Difference by GP characteristics

Differences in policy acceptance and experiences of GPs by GP characteristics are shown in S1 Table. The most notable difference was found for gender. Compared to female GPs, male GPs less often agreed with the policy that the regional public health services prescribed antiviral drugs instead of GPs, while they more often found the decision correct to make GPs responsible for the sampling of patients. Changes in the notification of influenza A(H1N1) virus infections (15 August-December 2009) was less often reported to be well communicated by male GPs, and male GPs also less often agreed with the decision to limit notification to hospitalised and deceased patients. Female GPs reported more often changes in their daily work due to the vaccination programme, and were less often satisfied with the compensation for vaccination.

### Practice assistants

#### Information provision and workload

Practice assistants were more positive about patient information provided by GPs than about information provided by the government, and positive about the information provided during the epidemic and vaccination period (Table 3). Almost all practice assistants experienced an increased workload due to the public attention paid to the influenza A(H1N1) virus and the hectic coverage in the media, as much as to the influenza A(H1N1) virus epidemic itself and vaccination. Eighty-six per cent of the assistants reported daily work to be compromised in the epidemic and vaccination period. Of the practice assistants that experienced extra time investment in telephonic calls about the influenza A(H1N1) virus, the median extra time investment per day was one to 1.5 hours.

**Table 3:** Policy acceptance and experiences of practice assistants during the influenza A(H1N1) pandemic.

Aspects during the influenza A(H1N1) pandemic	N total	N(strongly) agree	% (strongly) agree (95% CI)
*Information provision and workload(April-August 2009)*			
Enough information from government to inform patients	496	331	66.7% (62.6–70.9)
Enough information from the general practitioner(s) to inform patients	494	438	88.7% (85.9–91.5)
Information could be found about the influenza A(H1N1) virus when needed	494	403	81.6% (78.2–85.0)
The attention paid to the influenza A(H1N1) virus led to more telephone calls	498	493	99.0% (98.1–99.9)
The attention paid to influenza A(H1N1) led to an increased workload	498	479	96.2% (94.5–97.9)
Hectic coverage in the media on the influenza A(H1N1) virus led to an increased workload	499	489	98.0% (96.8–99.2)
Extra time invested in telephone calls about the influenza A(H1N1) virus	467	433	92.7% (90.4–95.1)
Average extra time per day when extra time invested	379	1 (median)	0.75–2 (interquartile range)
*Information provision and workload (October-December 2009)*			
Enough information from government to inform patients about vaccination	491	385	78.4% (74.8–82.1)
Enough information from the general practitioner(s) to inform patients about vaccination	501	478	95.4% (93.6–97.2)
Information could be found about vaccination when needed	494	444	89.9% (87.2–92.5)
The attention of the media to the side effects of vaccination led to more telephone calls	499	477	95.6% (93.8–97.4)
The flu epidemic and vaccinations led to an increased workload	501	498	99.4% (98.7–100)
During the epidemic and vaccinations, normal work was compromised	497	427	85.9% (82.9–89.0)
Extra time invested in telephone calls about the influenza A(H1N1) virus	490	482	98.4% (97.2–99.5)
Average extra time per day when extra time invested (median (interquartile range))	423	1.5 (median)	1–2 (interquartile range)

## Discussion

We showed that compliance with recommendations was not optimal, even in the event of the pandemic. In the case of the influenza A(H1N1) pandemic, the impact of non-compliance on health outcomes was not tremendous, since the pandemic proved to be mild. However, in the case of future, more severe pandemics, compliance with recommendations may be more crucial. This study provides some new insights of value for other countries.

### Strengths and limitations

This study is, to our knowledge, the first to report on the acceptance of the chosen policy and experiences of GPs and practice assistants during the influenza A(H1N1) pandemic. Our study has some limitations. First, data were collected ten months after the beginning of the influenza A(H1N1) pandemic and could have been subject to recall bias. This could have resulted in an underestimation of the impact of the influenza A(H1N1) pandemic. Second, non-responders were more often employed by another GP; therefore, GPs included in this study might not be representative of the Dutch general practice. However, additional analyses showed no differences in acceptance and experiences between GPs employed within a general practice and GPs working as free entrepreneurs, except regarding the moment when to take protective measures, which was more clear to GPs employed within a general practice (S1 Table).

### Comparison with existing literature and implications

Health care workers’ compliance with recommendations is of great importance for the delivery of health care during a pandemic. If health care workers do not comply with these measures, their infection chance increases, resulting in less available health care workers during a pandemic. This study showed that the personal protective measures advised for GPs until 10 June 2009 were believed to be difficult to implement in daily work, and almost half of the GPs reported not having executed these measures. Lack of knowledge and protective material did not seem to be the reason for the lack of feasibility and execution, as most GPs reported clear knowledge about when to take action and sufficient availability of personal protective material. In contrast, most frontline public health workers complied with the control measures in the Netherlands [3]. However, frontline public health workers were usually aware of the fact that they were seeing a suspected patient, while GPs might not always have been aware of patients’ infection status. As reported by other studies, protective measures are time consuming and sometimes difficult to organise (i.e. isolating infected patients) [4,5]. The advised protective measures for GPs were extensive in the Netherlands. In particular, cleaning after contact with suspected patients is time consuming, and provision of masks for suspected patients might not always be regarded as feasible in practice (e.g. practices with the reception desk in the waiting room for patients). It is important that suspected patients are provided with a face mask upon entering the practice, in order to prevent unnecessary virus spread. Future research should address the most feasible way in which these protective measures can be implemented in general practice.

Communication (of changes) of guidelines during a pandemic plays a major role in the implementation of recommendations. In general, GPs in the Netherlands were positive about the communication (of changes) during the influenza A(H1N1) pandemic and the collaboration with other involved organisations. This is in contrast with reports from Canada and the U.S. [6,8]. An explanation for the positive rating of communication and collaboration in the Netherlands could lie in the well-structured coordination for infectious diseases. In the Netherlands, the National Institute for Public Health and the Environment (RIVM) is, among others, responsible for the coordination and provision of information to patients and professionals in case of an infectious disease outbreak. During an outbreak, all involved actors work together in an outbreak management team and in managerial coordination meetings [10]. Other countries facing difficulties with regard to communication could learn from the Dutch approach.

Acceptance of the chosen policy is important for the implementation of recommendations, but sometimes acceptance of policy comes secondary to the overall containment strategy and monitoring of an infectious disease outbreak. Our study showed that the chosen primary policy was not accepted by all GPs. The low acceptance for the policy might be due to the relatively mild course of the pandemic, which became increasingly clear during the course of the pandemic. Alternatively, redirecting suspected patients to the regional public health service might be regarded as counterintuitive by GPs. However, for monitoring of the spread and impact of an infectious disease outbreak, it is important that all information is available to one organisation, which is easily managed by redirecting suspected patients to this organisation.

In accordance with previous studies, the additional vaccination programme resulted in an increased workload for GPs and practice assistants, and daily work was compromised [15]. Half of the GPs reported the compensation for additional tasks to be insufficient, as was also reported in a study in Canada [6]. In the case of the Netherlands, risk groups for vaccination could only be identified in general practice, which made GPs pre-eminently the group of health care workers to take care of the vaccination programme. The involvement of GPs is of great importance when considering future infectious disease outbreaks. However, for GPs to play a significant role in vaccination programmes in the future, increased financial compensation or availability of extra personnel may be necessary.

In conclusion, this study contributes to the adjustment of pandemic recommendations in general practice and raises issues that should be addressed before a next pandemic occurs. GPs play a pivotal role in the prevention and control of infectious disease spread, as patients with manifestation of illnesses and concerns regarding infectious diseases most likely present to a GP first. In the case of a pandemic, current recommendations with regard to protective measures do not seem to be sufficient. In incidental cases these extensive protective measures may be feasible, but in case of a pandemic with more infected patients this may not. Engaging GPs in the development of new operational scenarios for future pandemic is essential, in which a better understanding of practice differences among GPs in the Netherlands should facilitate this process. In addition, learning from the experiences of other countries is necessary to develop strategies to control new infectious diseases [16].

## Supporting Information

S1 Table Differences in policy acceptance and experiences of general practitioners during the influenza A(H1N1) pandemic by GP characteristics. (DOCX)
