# Supplementary material for: Experiences of General Practitioners and Practice Assistants during the Influenza A(H1N1) Pandemic in the Netherlands: A Cross-Sectional Survey
Source: PLoS One. 2015 Aug 27;10(8):e0135666. doi: 10.1371/journal.pone.0135666 (PMC4551952; doi:10.1371/journal.pone.0135666)
Supplement: S1 Table — (DOCX) [file pone.0135666.s001.docx]

**S1 Table. Differences in policy acceptance and experiences of general practitioners during the influenza A(H1N1) pandemic by GP characteristics**

| **Aspects during the influenza A(H1N1) pandemic** | **Statistically significant differences by GP characteristics (gender, age, function, practice type and urbanisation)** |
| --- | --- |
| *Information provision (April-December 2009)* |  |
| Information provided by the government for patients was explicit | GPs in group practices less often agreed (single-handed: 49%; duo 51%, group or health care centre 38%); p=0.046 |
| Information provided by the government for patients was well timed | Older GPs more often agreed (<40:47%; 40-49: 42%, 50-59: 60%, 60+: 65%); p=0.015 |
| *Protective measures (April-10 June 2009)* |  |
| Clear on when to take protection measure | GPs employed by another GP more often agreed (employed: 94%; free entrepreneur: 67%); p=0.026 |
|  | Younger GPs more often agreed (<40: 83%; 40-49:63%; 50-59: 67%; 60+: 65%); p=0.041 |
| Sufficient personal protection materials were in practice | GPs in duo practices less often agreed (single-handed:75%; duo: 65%, group or health care centre:78%);p=0.05 |
| *Mandatory notification of influenza A(H1N1) virus infections (April-15 August 2009)* |  |
| The notification was useful | GPs aged 50-59 years less often agreed (<40:53%; 40-49:51%; 50-59:35%; 60+:60%);p=0.004 |
| *Provision of antiviral drugs by the regional public health service (April-22 July 2009)* |  |
| Correct choice that the regional public health service prescribed antiviral drugs instead of the general practitioner | Single-handed GP less often agreed (single-handed:45%, duo:64%, group or health care centre:58%); p=0.046  Male GPs less often agreed (female:68%; male:50%); p=0.001 |
| *Changes in policy infection prevention and assessment and management of patients (June-July 2009)* |  |
| Good that general practitioners became responsible for the sampling of patients | Male GPs more often agreed (female:63%; male:73%); p=0.036 |
|  |  |
| *Changes in notification of influenza A(H1N1) virus infections (15 August-December 2009)* |  |
| Changes in notification or reporting were well communicated | Male GPs less often agreed (female:87%; male:76%);p=0.008 |
| Good decision to limit notification to hospitalised and deceased patients | Male GPs less often agreed (female:98%; male 92%);p=0.024 |
|  |  |
| *Policy on antiviral drugs for high-risk patients only (15 August-December 2009)* |  |
| Advice regarding the prescription of antiviral medicines was complete | GPs in duo practices less often agreed (single-handed:65%; duo:46%; group or healt care centre: 64%);p=0.007 |
|  |  |
| *Vaccination programme for influenza A(H1N1) in general practice (November 2009)* |  |
| During the vaccination rounds, not a lot of time for normal daily work | Male GPs less often agreed (female:79%; male: 66%);p=0.005 |
| During the epidemic, tasks were different compared with a regular influenza season | GPs in extremely urbanised areas more often agreed, and GPs in moderately urbanised areas less often agreed (extremely urbanised: 84%; strongly urbanised:75%; moderately urbanised: 61%; hardly urbanised:72%; not urbanised:75%);p=0.020  Male GPs less often agreed (female:84%; male: 67%); p<0.001 |
| Additional tasks due to vaccination were sufficiently compensated | Male GPs more often agreed (female:43%; male:55%); p=0.038 |
